# Worldwide Distribution of Major Clones of *Listeria monocytogenes*

**DOI:** 10.3201/eid1706.101778

**Published:** 2011-06

**Authors:** Viviane Chenal-Francisque, Jodie Lopez, Thomas Cantinelli, Valerie Caro, Coralie Tran, Alexandre Leclercq, Marc Lecuit, Sylvain Brisse

**Affiliations:** Author affiliations: Institut Pasteur, Paris, France (V. Chenal-Francisque, J. Lopez, T. Cantinelli, V. Caro, C. Tran, A. Leclercq, M. Lecuit, S. Brisse);; World Health Organization Collaborating Center for *Listeria*, Paris (V. Chenal-Francisque, A. Leclercq, M. Lecuit);; Inserm Avenir U604, Paris (M. Lecuit);; Université Paris Descartes, Paris (M. Lecuit)

**Keywords:** Listeria monocytogenes, genetic diversity, phylogeny, typing, epidemiology, geographic distribution, bacteria, dispatch

## Abstract

*Listeria* *monocytogenes* is worldwide a pathogen, but the geographic distribution of clones remains largely unknown. Genotyping of 300 isolates from the 5 continents and diverse sources showed the existence of few prevalent and globally distributed clones, some of which include previously described epidemic clones. Cosmopolitan distribution indicates the need for genotyping standardization.

*Listeria monocytogenes* is a foodborne pathogen that can cause listeriosis, a severe invasive infection in humans with a particularly high case-fatality rate. Listeriosis is a major public health concern in all world regions, with an increasing incidence in Europe, especially among elderly persons (*1**,**2*).

*L. monocytogenes* is genetically heterogeneous (*3**–**5*). To help epidemiologic investigation and to define clones, i.e., groups of genetically similar isolates descending from a common ancestor, a variety of typing methods have been used, including pulsed-field gel electrophoresis (*5**,**6*), single nucleotide polymorphism typing (*7*), and multiple housekeeping and virulence gene sequencing (*8**,**9*). Some clones implicated in multiple outbreaks have been defined as epidemic clones (EC) (*3**,**5**,**9**–**11*). ECI and ECIV have been described in several countries (*3**,**5*), but because of the lack of standardization of genotyping, a definition of clones is not widely accepted, and current knowledge on the global distribution of *L. monocytogenes* clones is virtually absent. Multilocus sequence typing (MLST) is a reference method for global epidemiology and population biology of bacteria, and its application to *L. monocytogenes* (*12*) effectively allows isolate comparisons across laboratories (www.pasteur.fr/mlst). The aim of this study was to investigate the global distribution of *L. monocytogenes* MLST-defined clones.

## The Study

Three hundred *L. monocytogenes* isolates were collected from different sources from 42 countries on 5 continents (Table A1). The isolates derived from 1) the collection of the World Health Organization Collaborating Center for *Listeria* and 2) the Seeliger *Listeria* Culture Collection. When available, up to 10 countries per continent were included. Only 1 isolate per documented outbreak was kept, and the isolates from a given country were selected from various sources, years, and serotypes. A total of 117 isolates were from humans, 107 from food, 28 from animals, 32 from the environment and vegetation, and 16 of undocumented origin. The relative proportion of isolates from distinct sources was similar among world regions (Table A1), except that no animal isolate was available from the Western Hemisphere and that the ratio of human to food isolates was lower from this continent.

Each isolate was hemolytic when streaked for isolation on blood agar. Genomic DNA was extracted by using Promega Wizard Genomic DNA purification kit (Promega, Madison, WI, USA). Serotype information was confirmed by PCR serogrouping (*13*). MLST was performed as described (*12*). Alleles and sequence types (STs) are publicly available at www.pasteur.fr/mlst. Clonal complexes (CC) were defined as groups of STs differing by only 1 gene from another member of the group (*12*) and were considered as clones. The θ estimator of the Fst statistic, which measures population differentiation, was determined on the basis of ST frequency by using FSTAT (www2.unil.ch/popgen/softwares/fstat.htm).

**Figure A1:**
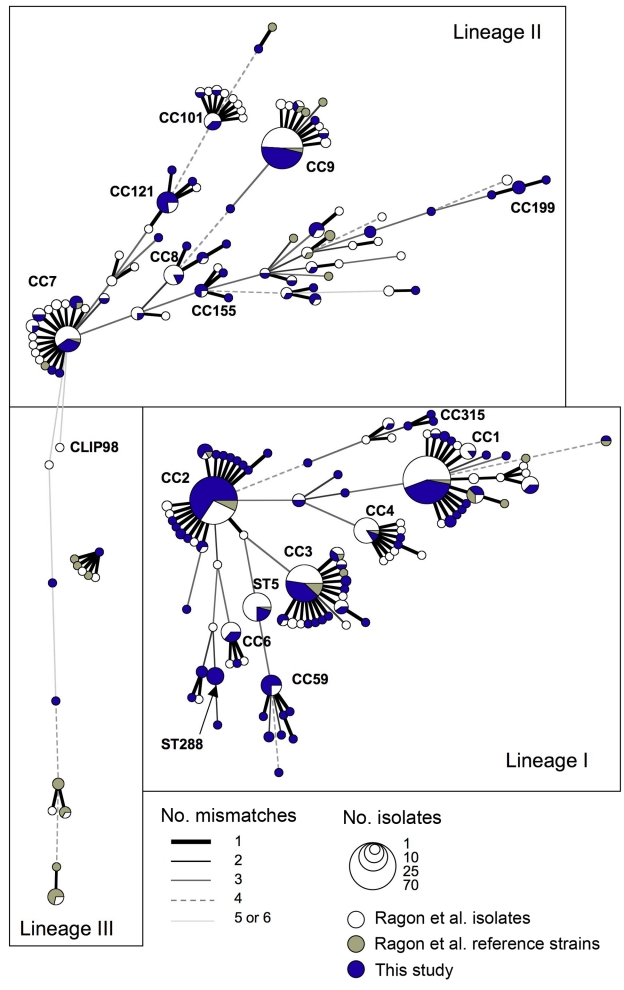
Genetic relationships among 660 *Listeria monocytogenes* isolates. The graph is a minimum spanning tree based on allelic profiles by using BioNumerics version 6.1 (Applied-Maths, Sint-Martens-Latem, Belgium). The 300 isolates of this study are in blue; the 360 *L. monocytogenes* isolates and reference strains of our earlier study (*12*) are in white and gray, respectively. Each circle represents a multilocus sequence typing genotype (ST), the size of which is related to the number of isolates (see legend). Clones, defined as clonal complexes [CC], are composed of groups of STs linked with a single gene difference, denoted as bold lines (see legend); clone number is indicated for the major clones. Links with >2 mismatches are unreliable because many alternatives may exist; links with 7 mismatches are not shown.

The 300 isolates represented 111 STs (diversity index 95.4%) grouped into 17 CCs (Figure A1). Phylogenetic analysis of the concatenated genes (not shown) indicated that 199, 98, and 3 isolates belonged to lineages I, II, and III, respectively (*12*). In lineage I, 3 CCs were highly prevalent: CC1 (47 isolates, serotype 4b), CC2 (64 isolates, 4b,) and CC3 (32 isolates, 1/2b). The remaining isolates of lineage I were of serotype 4b or 1/2b (Table). In lineage II, CC9 (28, all with serotype 1/2c, except one 1/2a isolate) was the most frequent, followed by CC7 (15 1/2a isolates). All other lineage II isolates had serotype 1/2a.

**Table:** Distribution of the major *Listeria monocytogenes* clonal complexes in lineages I and II among sources

Lineage or clonal complexes	No. (%), by source						Human/food ratio
	Total	Human	Food	Animal	Environment	Unknown	
Lineage I, total	199 (100)	88 (44)	60 (30)	16 (8)	24 (12)	11 (6)	1.60:1
CC1 (4b)	47 (100)	26 (55)	10 (21)	4 (9)	3 (6)	4 (9)	2.60:1
CC2 (4b)	64 (100)	36 (56)	13 (20)	6 (9)	5 (8)	4 (6)	2.77:1
CC3 (1/2b)	32 (100)	11 (34)	17 (53)	1 (3)	2 (6)	1 (3)	0.65:1
Other 4b	20 (100)	8 (40)	6 (30)	1 (5)	4 (20)	1 (5)	1.33:1
Other 1/2b	36 (100)	7 (19)	14 (39)	4 (11)	10 (28)	1 (3)	0.50:1
Lineage II, total	98 (100)	29 (30)	45 (46)	11 (11)	8 (8)	5 (5)	0.64:1
CC9 (1/2c)	28 (100)	7 (25)	14 (50)	2 (7)	3 (11)	2 (7)	0.50:1
Other, lineage II (1/2a)	70 (100)	22 (31)	31 (44)	9 (13)	5 (71)	3 (4)	0.71:1

Comparisons of populations from different sources (Table) showed a clear partitioning of genotypic diversity between clinical isolates on the one hand and food or environmental isolates on the other (θ = 0.033 and 0.050, respectively; p<0.0002). Consistent with common knowledge (*4**,**5*), and even though recent outbreaks in Canada and Austria/Germany were caused by 1/2a strains, isolates of serotype 4b were, compared with other serotypes, relatively more frequent in human cases than in food. This difference in source distribution was further demonstrated for individual clones because the human/food ratio of both CC1 (2.6) and CC2 (2.8) differed significantly from those of CC3 (0.65) and CC9 (0.5) (χ^2^ p<0.01 for the 4 comparisons).

A global distribution of *L. monocytogenes* clones was evident (Figure). Frequent clones were found in many countries (up to 30 countries for CC2; Table A1) and were globally distributed. Remarkably, CC1 and CC2 were predominant in all world regions except northern Africa for CC1 (Figure). CC3 ranked among the 4 most common clones in all regions, whereas CC9 ranked third in Europe and the Western Hemisphere. Altogether, these 4 clones represented 54 (50%) food isolates and 80 (68%) clinical isolates. Our results show that the same few clones account for a large fraction of nonepidemic *L. monocytogenes* isolates in distant world regions. However, continents and sources were not equally represented in our sample, and larger studies are needed to confirm our hypothesis that the clonal composition is similar across world regions and countries. Consistent with their cosmopolitan distribution, 15 of the 17 clones found herein (except CC199 and CC315, with only 6 and 3 isolates, respectively) included isolates from our previous analysis of 360 isolates, mostly from France (*12*).

## Conclusions

This study provides the first global view of *L. monocytogenes* clonal diversity. Our results clearly demonstrate the worldwide distribution and high prevalence of a few frequent clones in distinct world regions. In the current debate on the phylogeography of bacterial species (*14*), major *L. monocytogenes* clones clearly fit in the “everything is everywhere” group, as do other pathogens in the environment, e.g., *Pseudomonas aeruginosa* (*15*). Dispersal by human travel, animal or food trade, wild animal migration, or wind and dust all might contribute to the global diffusion of *L. monocytogenes* clones. However, finer phylogenetic resolution will possibly subdivide widespread MLST-defined clones into subclades that might exhibit phylogeographic partitioning and will better clarify the rate and patterns of strain dispersal.

Remarkably, some ECs correspond with highly prevalent clones. ECII, described relatively recently (*6*), and ECIII, involved in outbreaks from a single plant, correspond to 2 clones (CC6 and ST11, respectively [*12*]), that were rare herein (5 and 0 isolates, respectively), suggesting that both clones experienced particular conditions that favored their diffusion on specific occasions. In contrast, the outbreaks caused by ECI and ECIV, reference strains of which belong to CC1 and CC2, respectively (*12*), could have been favored by their high prevalence in sources. One important question for future research is whether ECs correspond entirely to MLST-defined clones (i.e., CCs) or whether, on the contrary, they represent a genotypic subset thereof. The cosmopolitan distribution of clones, which protects them against extinction resulting from local disturbances, further highlights the crucial need to standardize *L. monocytogenes* genotyping to improve global epidemiologic knowledge and monitoring of current emergence trends.

## Figures and Tables

**Figure:**
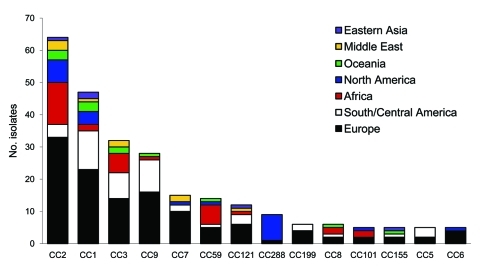
Number of isolates from 7 world regions where the most prevalent clones of *Listeria monocytogenes* are found.

## Appendix

**Table A1:** Sources and characteristics of *Listeria monocytogenes* isolates analyzed

Key	World region	Country	Year	Source	Lineage	CC*	ST†	Serotype‡
LM13415	Africa	Algeria	1985	Human	1	CC1	1	4b
LM13417	Africa	Algeria	1987	Human	1	CC2	2	4b
LM10976	Africa	Algeria	1988	Animal	1	CC2	2	4b
LM76305	Africa	Algeria	1998	Human	1	CC2	2	4b
LM05-00105	Africa	Algeria	Unknown	Human	1	CC2	272	4b
LM8818	Africa	Algeria	1988	Animal	2	CC21	21	1/2a
LM13419	Africa	Algeria	1987	Human	1	CC3	273	1/2b
LM68629	Africa	Algeria	1991	Human	2	CC8	8	1/2a
LM68625	Africa	Algeria	1992	Human	2	CC8	8	1/2a
LM86423	Africa	Madagascar	Unknown	Food	2	CC121	121	1/2a
LM77185	Africa	Morocco	Unknown	Food	1	CC1	1	4b
LM86525	Africa	Morocco	Unknown	Food	2	CC101	101	1/2a
LM07-00231	Africa	Morocco	Unknown	Food	1	CC2	2	4b
LM20800	Africa	Morocco	Unknown	Human	1	CC2	2	4b
LM75327	Africa	Morocco	Unknown	Human	1	CC2	277	4b
LM86505	Africa	Morocco	Unknown	Food	1	CC3	287	1/2b
LM75325	Africa	Morocco	Unknown	Human	1	CC3	3	1/2b
LM75328	Africa	Morocco	Unknown	Human	1	CC3	3	1/2b
LM18327	Africa	Morocco	1990	Human	1	CC3	41	1/2b
LM77479	Africa	Morocco	Unknown	Vegetal	1	CC3	68	1/2b
LM77195	Africa	Morocco	Unknown	Food	1	CC59	282	1/2b
LM85273	Africa	Morocco	2000	Environment	1	CC59	285	1/2b
LM86481	Africa	Morocco	Unknown	Food	1	CC59	286	1/2b
LM07-00586	Africa	Morocco	Unknown	Food	1	CC59	59	1/2b
LM89984	Africa	Senegal	Unknown	Unknown	1	CC2	145	4b
LM09-00263	Africa	Senegal	2007	Food	1	CC59	59	1/2b
LM09-00264	Africa	Senegal	2009	Food	1	CC59	59	1/2b
LM09-00277	Africa	Tunisia	2007	Food	2	CC101	101	1/2a
LM58204	Africa	Tunisia	1994	Human	1	CC2	2	4b
LM09-00290	Africa	Tunisia	2005	Human	1	CC2	2	4b
LM09-00291	Africa	Tunisia	2007	Human	1	CC2	2	4b
LM20490	Africa	Tunisia	Unknown	Environment	1	CC2	2	4b
LM71962	Africa	Tunisia	Unknown	Food	1	CC2	276	4b
LM42224	Africa	Tunisia	1993	Human	2	CC9	9	1/2c
LM21416	Eastern Asia	China	1991	Human	1	CC1	1	4b
LM12980	Eastern Asia	China	1987	Unknown	1	CC1	243	4b
LM22261	Eastern Asia	China	1992	Food	2	CC121	121	1/2a
LM21413	Eastern Asia	China	1991	Human	2	CC155	155	1/2a
LM21436	Eastern Asia	China	Unknown	Food	1	CC2	2	4b
SLCC875	Eastern Asia	Russia	1948	Food	2		126	1/2a
LM12872	Europe	Austria	1986	Human	2	CC14	91	1/2a (s)
LM8792	Europe	Austria	1987	Food	2	CC18	18	1/2a (s)
LM83291	Europe	Austria	2000	Human	1	CC195	195	1/2b (s)
LM8789	Europe	Austria	1987	Food	2	CC199	199	1/2a (s)
LM8800	Europe	Austria	1988	Food	2	CC199	199	1/2a (s)
LM8806	Europe	Austria	1988	Food	2	CC199	199	1/2a (s)
LM8785	Europe	Austria	1987	Food	1	CC2	2	4b (s)
LM8736	Europe	Austria	1986	Food	1	CC3	3	1/2b (s)
LM8742	Europe	Austria	1986	Food	2	CC9	9	1/2c (s)
LM8796	Europe	Austria	1988	Food	2	CC9	9	1/2c
LM11971	Europe	Belgium	1987	Animal	2		177	1/2a
LM07-00578	Europe	Belgium	1990	Food	2		222	1/2a
LM17434	Europe	Belgium	1988	Food	1	CC1	10	4b (s)
LM07-00581	Europe	Belgium	1990	Food	2	CC121	121	1/2a
LM17416	Europe	Belgium	1988	Food	2	CC18	18	1/2a (s)
LM23000	Europe	Belgium	1990	Food	2	CC199	230	1/2a (s)
LM17413	Europe	Belgium	1988	Human	1	CC2	2	4b (s)
LM17559	Europe	Belgium	1989	Human	1	CC2	2	4b (s)
LM17571	Europe	Belgium	1989	Human	1	CC3	3	1/2b (s)
LM83769	Europe	Belgium	1999	Human	1	CC4	4	4b (s)
LM11970	Europe	Belgium	1986	Human	1	CC59	241	1/2b (s)
LM17431	Europe	Belgium	1988	Food	1	CC59	59	1/2b (s)
SLCC2298	Europe	Bulgaria	1965	Animal	1	CC1	1	4b (s)
LM83919	Europe	Croatia	<2000	Animal	1	CC2	2	4b (s)
LM80112	Europe	Croatia	<2000	Animal	1	CC3	235	1/2b (s)
LM78358	Europe	Croatia	<1999	Animal	2	CC37	234	1/2a (s)
LM83916	Europe	Croatia	<2000	Animal	2	CC8	120	1/2a (s)
LM07-00288	Europe	Czech Republic	<2007	Unknown	2	CC14	14	1/2a
LM69539	Europe	Czech Republic	1995	Environment	1	CC195	195	1/2b
LM05-01034	Europe	Czech Republic	<2005	Unknown	1	CC2	145	4b
LM69522	Europe	Czech Republic	1995	Food	1	CC5	5	1/2b
LM05-01033	Europe	Czech Republic	<2005	Unknown	2	CC7	12	1/2a
LM82202	Europe	Denmark	1999	Human	1	CC1	79	4b (s)
SLCC1531	Europe	Denmark	1962	Human	1	CC2	2	4b
SLCC1686	Europe	Denmark	1963	Human	1	CC2	2	4b (s)
LM10791	Europe	Denmark	1988	Human	1	CC2	2	4b
SLCC1533	Europe	Denmark	1962	Human	1	CC2	257	4b (s)
SLCC2399	Europe	Denmark	1966	Animal	1	CC2	48	4b (s)
LM10378	Europe	Denmark	1988	Food	1	CC59	59	1/2b
LM12832	Europe	Denmark	1989	Human	2	CC7	7	1/2a
SLCC69	Europe	Denmark	1937	Human	2	CC7	98	1/2a
LM14228	Europe	Denmark	1989	Food	2	CC9	9	1/2a
LM15178	Europe	Finland	1989	Human	2	CC121	121	1/2a
LM15142	Europe	Finland	1986	Human	1	CC2	2	4b (s)
LM8013	Europe	Finland	1987	Food	1	CC3	3	1/2b
LM15208	Europe	Finland	1988	Environment	1	CC3	3	1/2b
LM8017	Europe	Finland	1974	Human	1	CC315	253	4b
LM8169	Europe	Finland	1987	Animal	1	CC6	254	4b
LM15256	Europe	Finland	1988	Animal	2	CC7	227	1/2a
LM8077	Europe	Finland	1987	Food	2	CC7	7	1/2a
LM15183	Europe	Finland	1989	Human	2	CC9	9	1/2c
SLCC1612	Europe	France	1963	Human	1	CC1	73	4b
LM23187	Europe	France	1992	Food	1	CC2	2	4b
SLCC546	Europe	France	1956	Human	1	CC3	117	1/2b
LM15262	Europe	France	1990	Human	1	CC3	228	1/2b (s)
SLCC206	Europe	France	1950	Food	1	CC315	102	4b
LM12519	Europe	France	1989	Human	1	CC4	242	4b (s)
LM21131	Europe	France	1992	Food	1	CC4	4	4b (s)
LM62444	Europe	France	1995	Food	2	CC7	231	1/2a
LM12485	Europe	France	1988	Food	2	CC9	9	1/2c
LM06-00139	Europe	France	2006	Environment	2	CC9	9	1/2c
LM71759	Europe	Germany	<1996	Unknown	1	CC1	1	4b (s)
LM65123	Europe	Germany	1994	Human	1	CC1	1	4b
LM65127	Europe	Germany	1995	Human	1	CC1	1	4b
SLCC39	Europe	Germany	1953	Human	2	CC101	113	1/2a
SLCC1023	Europe	Germany	1960	Animal	2	CC101	143	1/2a
LM82806	Europe	Germany	<2000	Food	2	CC121	121	1/2a (s)
SLCC2	Europe	Germany	1953	Human	1	CC2	145	4b
LM74698	Europe	Germany	<1997	Unknown	1	CC2	2	4b
LM82901	Europe	Germany	<2000	Human	2	CC21	21	1/2a (s)
LM18662	Europe	Germany	1990	Human	1	CC3	3	1/2b
LM82900	Europe	Germany	<2000	Animal	1	CC5	5	1/2b (s)
LM71758	Europe	Germany	1996	Unknown	2	CC7	12	1/2a (s)
SLCC1	Europe	Germany	1951	Human	2	CC7	23	1/2a
SLCC2864	Europe	Germany	1968	Food	2	CC7	7	1/2a (s)
LM78008	Europe	Germany	<1999	Unknown	2	CC9	9	1/2c (s)
LM70960	Europe	Greece	<1996	Animal	1		191	1/2b (s)
LM93187	Europe	Greece	<2003	Human	1	CC1	1	4b (s)
LM70927	Europe	Greece	<1996	Animal	1	CC1	252	4b (s)
LM93191	Europe	Greece	<2003	Human	2	CC155	237	1/2a (s)
LM70940	Europe	Greece	<1996	Animal	1	CC2	2	4b (s)
LM70290	Europe	Greece	<1996	Human	1	CC4	251	4b (s)
LM67774	Europe	Greece	<1995	Environment	1	CC6	6	4b
LM67783	Europe	Greece	<1995	Food	1	CC6	6	4b
LM67781	Europe	Greece	<1995	Food	2	CC9	115	1/2c
LM70956	Europe	Greece	<1996	Animal	2	CC9	9	1/2c (s)
LM9414	Europe	Italy	1988	Animal	1		191	1/2b
LM15852	Europe	Italy	1990	Human	1	CC1	1	4b
LM7616	Europe	Italy	1987	Human	1	CC1	252	4b
LM13394	Europe	Italy	1989	Food	2	CC121	121	1/2a
LM41103	Europe	Italy	1993	Food	2	CC18	18	1/2a
LM61388	Europe	Italy	1994	Food	1	CC2	246	4b
LM86834	Europe	Italy	<2001	Food	1	CC3	3	1/2b (s)
LM68619	Europe	Italy	1995	Human	2	CC8	120	1/2a
LM07-01172	Europe	Italy	2003	Animal	2	CC9	9	1/2c
LM06-00810	Europe	Italy	2005	Environment	2	CC9	9	1/2c
SLCC1457	Europe	The Netherlands	1962	Animal	1	CC1	1	4b (s)
LM07-01127	Europe	The Netherlands	<2007	Food	1	CC6	6	4b
LM07-01124	Europe	The Netherlands	<2007	Human	2	CC9	223	1/2c
SLCC2280	Europe	Poland	1965	Human	2	CC21	238	1/2a
LM08-00013	Europe	Portugal	2000	Human	1		224	1/2b
LM08-00002	Europe	Portugal	2003	Human	1		54	4b
LM70125	Europe	Portugal	<1996	Environment	1	CC1	1	4b (s)
LM84790	Europe	Portugal	<2000	Environment	1	CC1	255	4b (s)
LM08-01095	Europe	Portugal	<2005	Human	2	CC121	121	1/2a
LM70124	Europe	Portugal	<1996	Environment	2	CC121	121	1/2a (s)
LM08-00024	Europe	Portugal	2004	Human	2	CC16	16	1/2a
LM76641	Europe	Portugal	1998	Food	1	CC2	2	4b (s)
LM71637	Europe	Portugal	<1996	Food	1	CC288	233	1/2b (s)
LM70140	Europe	Portugal	<1996	Food	1	CC3	228	1/2b (s)
LM70139	Europe	Portugal	<1996	Food	1	CC3	3	1/2b (s)
LM08-00016	Europe	Portugal	1998	Human	1	CC59	59	1/2b
LM71634	Europe	Portugal	<1996	Food	2	CC9	9	1/2c (s)
LM05-01099	Europe	Portugal	<2005	Human	2	CC9	9	1/2c
LM87422	Europe	Spain	2000	Human	1	CC1	256	4b (s)
LM13478	Europe	Spain	<1989	Animal	1	CC2	2	4b
LM13479	Europe	Spain	<1989	Human	1	CC2	2	4b
LM13608	Europe	Spain	1989	Food	1	CC2	2	4b
LM59597	Europe	Spain	1991	Human	1	CC2	2	4b
LM38931	Europe	Spain	1992	Human	1	CC2	2	4b
LM65719	Europe	Spain	1995	Human	1	CC2	2	4b
LM9134	Europe	Spain	1988	Food	2	CC9	9	1/2c
LM68804	Europe	Sweden	1973	Human	1	CC1	1	4b
LM68807	Europe	Sweden	1974	Human	1	CC1	1	4b
LM68853	Europe	Sweden	1995	Environment	1	CC1	1	4b
LM68828	Europe	Sweden	1987	Human	1	CC1	248	4b
LM68833	Europe	Sweden	1988	Human	1	CC2	2	4b
LM68834	Europe	Sweden	1994	Human	1	CC2	2	4b
LM68802	Europe	Sweden	1975	Human	1	CC2	247	4b
LM68856	Europe	Sweden	1991	Human	1	CC3	3	1/2b
LM68846	Europe	Sweden	1994	Environment	1	CC315	249	4b
LM68865	Europe	Sweden	Unknown	Food	1	CC315	250	4b
SLCC792	Europe	Switzerland	1958	Human	1		240	4b (s)
SLCC2570	Europe	Switzerland	1967	Animal	3		201	L
LM51770	Europe	Switzerland	1994	Food	1	CC1	1	4b (s)
SLCC1849	Europe	Switzerland	1964	Animal	1	CC1	258	4b
SLCC243	Europe	Switzerland	1954	Animal	2	CC155	239	1/2a
LM60557	Europe	Switzerland	1994	Animal	2	CC18	18	1/2a (s)
LM74493	Europe	Switzerland	1997	Environment	1	CC2	2	4b (s)
LM51415	Europe	Switzerland	1993	Human	1	CC2	245	4b (s)
SLCC796	Europe	Switzerland	1959	Animal	1	CC2	259	4b (s)
LM16587	Europe	Switzerland	1990	Animal	1	CC59	59	1/2b (s)
LM14569	Europe	Turkey	1989	Food	2		226	1/2a (s)
LM14571	Europe	Turkey	1988	Food	2		226	1/2a (s)
LM14574	Europe	Turkey	1988	Food	2		226	1/2a (s)
LM13903	Europe	Turkey	1989	Food	1	CC3	225	1/2b (s)
LM13904	Europe	Turkey	<1989	Food	1	CC3	3	1/2b (s)
SLCC125	Europe	United Kingdom	1954	Human	1		54	4b
LM10453	Europe	United Kingdom	<1988	Food	1	CC1	1	4b
LM42629	Europe	United Kingdom	1993	Unknown	1	CC1	73	4b
SLCC1419	Europe	United Kingdom	1962	Human	1	CC2	2	4b (s)
SLCC1616	Europe	United Kingdom	1963	Human	1	CC2	2	4b (s)
SLCC2524	Europe	United Kingdom	1966	Human	1	CC2	2	4b (s)
LM8720	Europe	United Kingdom	1988	Human	1	CC2	2	4b (s)
LM24902	Europe	United Kingdom	1992	Food	2	CC7	7	1/2a
SLCC21	Europe	United Kingdom	1935	Human	2	CC9	122	1/2c
LM19700	Middle East	Iraq	Unknown	Food	1	CC2	145	4b
LM19695	Middle East	Iraq	Unknown	Food	1	CC3	66	1/2b
LM71350	Middle East	Iran	Unknown	Food	2	CC7	12	1/2a (s)
LM89979	Middle East	Israel	Unknown	Human	1	CC2	2	4b (s)
LM89980	Middle East	Israel	Unknown	Food	1	CC3	68	1/2b (s)
LM56346	Middle East	Israel	1993	Animal	2	CC7	7	1/2a
LM77966	Middle East	Qatar	Unknown	Human	1	CC1	1	4b (s)
LM80419	Middle East	Qatar	Unknown	Food	2	CC121	236	1/2a (s)
LM80415	Middle East	Qatar	Unknown	Food	1	CC2	2	4b (s)
SLCC665	North America	Canada	1958	Human	1	CC1	1	4b
SLCC183	North America	Canada	1954	Human	2	CC101	101	1/2a
SLCC907	North America	Canada	1953	Food	2	CC14	160	1/2a
SLCC537	North America	Canada	Unknown	Human	1	CC2	257	4b
SLCC357	North America	Canada	1950	Human	1	CC2	290	4b
SLCC236	North America	Canada	1954	Human	2	CC21	22	1/2a
SLCC100	North America	Canada	1951	Human	2	CC7	98	1/2a
LM88502	North America	Mexico	1999	Vegetal	1		32	4b
LM88513	North America	Mexico	1999	Environment	1	CC2	2	4b
LM88519	North America	Mexico	2000	Vegetal	1	CC2	2	4b
LM88520	North America	Mexico	2000	Environment	1	CC2	48	4b
LM88454	North America	Mexico	1999	Environment	1	CC288	288	1/2b
LM88455	North America	Mexico	1999	Vegetal	1	CC288	288	1/2b
LM88458	North America	Mexico	1999	Environment	1	CC288	288	1/2b
LM88461	North America	Mexico	1999	Environment	1	CC288	288	1/2b
LM88469	North America	Mexico	1999	Vegetal	1	CC288	288	1/2b
LM88491	North America	Mexico	2000	Vegetal	1	CC288	288	1/2b
LM88493	North America	Mexico	2000	Environment	1	CC288	288	1/2b
LM88500	North America	Mexico	2000	Environment	1	CC288	288	1/2b
LM88501	North America	Mexico	1999	Environment	1	CC6	6	4b
SLCC422	North America	United States	1956	Food	3		294	L
SLCC63	North America	United States	1933	Human	1	CC1	119	4b
SLCC65	North America	United States	1933	Human	1	CC1	119	4b
SLCC83	North America	United States	1937	Food	1	CC1	119	4b
SLCC299	North America	United States	1955	Human	1	CC2	2	4b
SLCC527	North America	United States	1957	Human	1	CC2	291	4b
SLCC459	North America	United States	Unknown	Unknown	1	CC59	292	1/2b
LM13007	Oceania	Australia	<1989	Unknown	1	CC2	2	4b
LM21475	Oceania	Australia	<1992	Environment	2	CC204	204	1/2a
LM19717	Oceania	New Zealand	1991	Food	2		229	1/2a
LM11309	Oceania	New Zealand	1987	Human	1	CC1	1	4b
LM18429	Oceania	New Zealand	1991	Food	1	CC1	1	4b
LM70327	Oceania	New Zealand	1995	Human	1	CC1	248	4b (s)
LM18427	Oceania	New Zealand	1991	Human	2	CC155	155	1/2a
LM70320	Oceania	New Zealand	1995	Human	1	CC2	2	4b (s)
LM18354	Oceania	New Zealand	1989	Human	1	CC2	244	4b
LM13044	Oceania	New Zealand	1981	Human	1	CC3	3	1/2b (s)
LM18357	Oceania	New Zealand	1990	Human	1	CC3	3	1/2b
LM40964	Oceania	New Zealand	1993	Human	1	CC59	59	1/2b
LM62719	Oceania	New Zealand	1994	Human	2	CC8	232	1/2a
LM11308	Oceania	New Zealand	1988	Human	2	CC9	9	1/2c
LM66720	South and Central America	Argentina	1995	Unknown	1	CC1	1	4b
LM77778	South and Central America	Argentina	1998	Food	1	CC1	1	4b
LM77638	South and Central America	Argentina	1997	Food	2	CC199	283	1/2a
LM69877	South and Central America	Argentina	Unknown	Human	1	CC2	2	4b
LM80547	South and Central America	Argentina	1999	Food	1	CC3	117	1/2b
LM75973	South and Central America	Argentina	1997	Food	1	CC3	3	1/2b
LM77713	South and Central America	Argentina	1998	Food	1	CC3	3	1/2b
LM89775	South and Central America	Argentina	2001	Food	1	CC3	3	1/2b
LM80503	South and Central America	Argentina	1999	Food	1	CC3	66	1/2b
LM78899	South and Central America	Argentina	1998	Food	2	CC9	9	1/2c
LM80539	South and Central America	Argentina	1999	Food	2	CC9	9	1/2c
LM89220	South and Central America	Argentina	2001	Food	2	CC9	9	1/2c
LM16678	South and Central America	Brazil	1989	Human	1		218	4b
LM16713	South and Central America	Brazil	1989	Food	3		293	L
LM71345	South and Central America	Brazil	Unknown	Human	1	CC1	73	4b
LM71338	South and Central America	Brazil	Unknown	Vegetal	2	CC121	275	1/2a
LM71346	South and Central America	Brazil	Unknown	Human	1	CC195	195	1/2b (s)
LM16722	South and Central America	Brazil	1990	Human	1	CC195	274	1/2b
LM16728	South and Central America	Brazil	Unknown	Vegetal	2	CC9	9	1/2c
LM77137	South and Central America	Chile	Unknown	Food	1		279	4b
LM70662	South and Central America	Chile	Unknown	Human	1	CC1	1	4b
LM90287	South and Central America	Chile	Unknown	Food	1	CC1	1	4b
LM77135	South and Central America	Chile	Unknown	Human	1	CC1	278	4b
LM77141	South and Central America	Chile	Unknown	Food	1	CC3	280	1/2b
LM77152	South and Central America	Chile	Unknown	Food	1	CC3	281	1/2b
LM77145	South and Central America	Chile	Unknown	Food	1	CC5	5	1/2b
LM77157	South and Central America	Chile	Unknown	Food	1	CC5	5	1/2b
LM90283	South and Central America	Chile	Unknown	Food	1	CC5	5	1/2b
LM77123	South and Central America	Chile	Unknown	Human	2	CC7	7	1/2a
LM90291	South and Central America	Chile	Unknown	Food	2	CC8	289	1/2a
LM70681	South and Central America	Chile	Unknown	Food	2	CC9	9	1/2c
LM77132	South and Central America	Chile	Unknown	Human	2	CC9	9	1/2c
LM77150	South and Central America	Chile	Unknown	Food	2	CC9	9	1/2c
LM84996	South and Central America	Colombia	1997	Food	1	CC1	1	4b
LM85003	South and Central America	Colombia	1998	Food	1	CC1	248	4b
LM85008	South and Central America	Colombia	1999	Food	2	CC121	121	1/2a
LM06-01614	South and Central America	Colombia	Unknown	Environment	2	CC199	199	1/2a
LM85020	South and Central America	Colombia	1999	Food	1	CC2	2	4b
LM85058	South and Central America	Colombia	2000	Food	1	CC2	2	4b
LM06-01589	South and Central America	Colombia	Unknown	Food	1	CC2	2	4b
LM84999	South and Central America	Colombia	1998	Food	1	CC59	59	1/2b
LM85044	South and Central America	Colombia	2000	Food	2	CC7	7	1/2a
LM06-01598	South and Central America	Colombia	Unknown	Food	1	CC87	87	1/2b
LM84995	South and Central America	Colombia	1997	Food	2	CC9	9	1/2c
LM06-01615	South and Central America	Colombia	Unknown	Food	2	CC9	9	1/2c
LM06-00547	South and Central America	French Guiana	2006	Human	1	CC1	1	4b
LM78969	South and Central America	French Guiana	1999	Vegetal	2	CC121	121	1/2a
LM17308	South and Central America	French Guiana	1990	Human	2	CC155	155	1/2a
LM16656	South and Central America	Guatemala	Unknown	Human	1	CC1	1	4b
LM18311	South and Central America	Guatemala	Unknown	Human	1	CC1	1	4b
LM18351	South and Central America	Guatemala	Unknown	Human	1	CC1	1	4b
LM84817	South and Central America	Peru	Unknown	Unknown	1		284	4b
LM84813	South and Central America	Peru	Unknown	Unknown	1	CC3	3	1/2b
LM84810	South and Central America	Peru	Unknown	Unknown	2	CC9	9	1/2c

*Strains with no clonal complex (CC) assignation correspond to singletons, i.e. genotypes that are not closely related to any other genotype. †ST, sequence type. ‡Determined by classical serotyping (s) or by PCR serotyping; for the latter, the indicated serotype corresponds to the most common serotype of the PCR group.
